# Decolonising global health in Africa: research agendas in public health, law, and human rights: David Patterson

**DOI:** 10.1093/eurpub/ckac131.100

**Published:** 2022-10-25

**Authors:** D Patterson, M Woldeyes, N Schuck, E Henricson, N Njuguna, M Oduor

**Affiliations:** 1 Groningen Centre for Health Law, University of Groningen, Groningen, Netherlands; 2 Faculty of Law, University of Nairobi, Nairobi, Kenya; 3 Faculty of Law, Moi University, Eldoret, Kenya

## Abstract

**Background:**

In recent years the Groningen Centre for Health Law (‘GCHL’ - formerly the Global Health Law Groningen Research Centre), Netherlands, has held annual summer schools on global health, law, and human rights. Responding to calls to decolonise global health (Fofana, 2021), in February 2022 GCHL convened an online academic colloquium to explore the issues in Africa. Panellists and discussants comprised leading African academics and advocates for public health, law, and human rights.

**Objectives:**

1. Identify priority current and emerging issues in global health, law, and human rights in the African region with, where possible, reference to the climate crisis.

2. Explore opportunities for identifying academic institutions, networks, and researchers working these issues across Africa.

3. Identify opportunities to support collaboration between institutions, networks and researchers and other actors to address the issues identified across the region

**Results:**

Top public health issues identified for further research included: public health law frameworks in Africa; One Health and climate change; inequality in the distribution of the determinants of health and disease; international trade and public health; the right to benefit from scientific progress (e.g. in accessing vaccines for COVID-19); gender-based violence; public health and agri-food systems; noncommunicable diseases; healthy diets; poverty; mental health; social protection; and plastic pollution. The first meeting of the network on health, law and human rights in Africa was held in May 2022. The second academic colloquium was held in July 2022, co-hosted with Moi University and the University of Nairobi, Kenya.

**Conclusions:**

Public health and legal academics in Africa are ready to engage systematically with European partners to address key health-related law and human rights issues of global interest. Research agendas should reflect African priorities, and collaboration should be led by African institutions.

**Key messages:**

• Capacity must be built to understand the links between public health, law and human rights in Africa.

• Collaboration with European institutions to build capacity in public health, law and human rights is welcome, however priorities should be identified by - and responses led by - African academics.

